# Cell Condensation Triggers the Differentiation of Osteoblast Precursor Cells to Osteocyte-Like Cells

**DOI:** 10.3389/fbioe.2019.00288

**Published:** 2019-10-23

**Authors:** Jeonghyun Kim, Taiji Adachi

**Affiliations:** Biomechanics Laboratory, Institute for Frontier Life and Medical Sciences, Kyoto University, Kyoto, Japan

**Keywords:** spheroids, differentiation, osteocyte, osteoblast, rotatory culture, cell condensation

## Abstract

Though the three-dimensional (3D) *in vitro* culture system has received attention as a powerful tool for conducting biological research, *in vitro* bone formation and osteocyte differentiation studies have mostly been based on results obtained using two-dimensional (2D) culture systems. Here, we introduced a rotatory culture system to fabricate 3D spheroids, using mouse osteoblast precursor cells. These spheroids, incubated for 2 days without chemical induction by osteogenic supplements, exhibited notably up-regulated osteocyte marker levels; osteoblast marker levels were down-regulated, as compared to those of the conventional 2D monolayer model. The cell condensation achieved with the 3D spheroid structure triggered a greater level of differentiation of osteoblast precursor cells into osteocyte-like cells than that observed during chemical induction. Our study might imply that osteoblasts proliferate and become condensed at the targeted bone remodeling site, because of which osteoblasts achieved the capability to differentiate into osteocytes *in vivo*.

## Introduction

Three-dimensional (3D) culture systems known as organoids have recently been in focus, as they have been used to recapitulate the morphogenesis and functioning of various types of organs (Sasai, 2013; Rossi et al., 2018), such as the small intestine (Spence et al., 2011), liver (Takebe et al., 2013), stomach (McCracken et al., 2014), and lungs (Dye et al., 2015). The 3D structure of cells inducing the appropriate cell-cell interactions during the organ formation process has become known; hence, the demand for the generation of a 3D *in vitro* culture system by researchers studying human development, disease, and drug screening has increased (Rossi et al., 2018). However, the structural effects and configurations of cells in the 3D culture system, and especially cellular behavior, including differentiation capability, are not fully understood yet. Although a conventional two-dimensional (2D) culture system has greatly enabled us to understand cellular behavior, including gene expression and homeostasis, it might alter several intracellular signaling pathways, as compared to those present *in vivo*, thereby causing distinct biological outcomes. Hence, a more detailed study using the 3D culture system would be required to understand cellular behavior in complex native structures.

While the 3D culture system is regarded as a promising tool for performing *in vitro* biological studies, the introduction of the 3D model is also thought to have influenced the study of *in vitro* bone formation. The bone is composed of mineralized collagen fibrils induced via the formation of apatite crystals (Nair et al., 2013), and it is also known as a dynamic tissue that undergoes remodeling with osteoclasts and osteoblasts throughout the lifespan of a mammal (Weatherholt et al., 2012). Osteocytes comprise ~95% of bone cells that are embedded inside the mineralized bone matrix (Adachi et al., 2009; Bonewald, 2011). Due to the difficulty in retaining the osteocyte-likeness *in vitro* after osteocyte isolation, *in vitro* models utilizing osteocytes are fewer in number, whereas osteoblasts have been utilized as a surrogate. However, current *in vitro* bone formation and osteocyte differentiation studies have mostly been carried out with the 2D model, using the chemical induction process. The role of chemical supplements, such as ascorbic acid and β-glycerophosphate in the osteogenic differentiation process was successfully revealed with the use of this model (Malaval et al., 1994; Coelho and Fernandes, 2000; Buttery et al., 2004). Moreover, the conventional methods allowed osteoprogenitor cells to induce osteogenic differentiation over 3–4 weeks (Quarles et al., 1992; Wang et al., 1999). As a result of this long-term cultivation of osteoprogenitor cells, the proliferated cells formed a highly localized pile of confluent cells, which resulted in the bone nodule having a 3D dome-shaped structure (Bhargava et al., 1988; Kawai et al., 2019). Inside the bone nodule, the cells are induced to differentiate into osteoblasts, and these cells secreted a highly organized collagen matrix and further mineralized the deposited extracellular matrix (ECM), including alkaline phosphatase (ALP). Furthermore, osteocyte-like cells were observed inside this *in vitro* bone nodule (Kawai et al., 2019). These *in vitro* results, however, are yet to sufficiently mimic the *in vivo* bone formation with regard to the level of differentiation and induction time (Blair et al., 2017). Hence, a paradigm shift is required in a new *in vitro* osteocyte model, such as the 3D culture system. The development of the new *in vitro* 3D osteocyte culture model is expected to provide new insights into the biology of osteocytes and the utilization of this information to achieve well-organized bone formation. Apart from its application *in vitro*, the 3D osteocyte model is also predicted to become a potential tool for tissue-engineered regenerative medicine, owing to the ability to transplant them. We hereby postulate that the cell condensation achieved with the 3D structure provides an appropriate cell-ECM interaction for cells and further plays a significant role in osteogenic differentiation.

In this study, it is proposed that the cell condensation in the 3D culture system efficiently induced osteogenic differentiation. Particularly, we fabricated 3D bone spheroids using pre-osteoblast cells under rotatory culture conditions, without introducing any chemical supplements. By using this model, we evaluated the differentiation capacity of the spheroids and further attempted to highlight the driving force for triggering *in vitro* osteogenic differentiation.

## Materials and Methods

### Cell Culture

In this study, we utilized the murine pre-osteoblast cell line MC3T3-E1. Cells were cultured in MEM-α (Gibco), consisting of 10% fetal bovine serum (Gibco), and 1% antibiotic-antimycotic (Gibco) solution in a humidified incubator at 37°C, in the presence of 5% CO_2_. We carried out passaging when the confluency of the cells became up to 80–90%. To prepare an osteogenic induction medium, we subcultured cells with osteogenic supplements containing 50 μg/ml ascorbic acid and 10 mM β-glycerophosphate. To prepare the 2D monolayer sample, 200,000 cells were subcultured on a 35 mm culture dish (cell density: 208 cells/mm^2^), to make it fully confluent after a 2-days incubation period.

### Fabrication of Spheroids

We fabricated cell spheroids using MC3T3-E1 cells, by optimizing the protocol described by Furukawa et al. (2001). First, 1,000,000 cells were subcultured with the culture medium in a 6-well ultra-low attachment plate (Corning Coaster) (cell density: 1,040 cells/mm^2^). The culture plate containing the subcultured cells was then subjected to a rotational culture process at 70 rpm for 8 h, in a humidified incubator at 37°C, in the presence of 5% CO_2_. The spheroids formed were further incubated in a stationary culture process for 2–4 days.

### Real Time-PCR

The samples were collected and lysed in 1 ml of Isogen II (Nippon Gene) for RNA extraction. After adding 50 μl of p-Bromoanisole (Nacalai Tesque), the samples were centrifuged at 12,000 g for 10 min at 4°C. The supernatants were separated from the mixture and transferred to new microtubes, into which the same amounts of 70% ethanol were added. After mixing the tube well, the mixtures were transferred into the spin cartridge (PureLink RNA Mini kit; Invitrogen). The samples were subjected to the centrifugation process, followed by washing and drying, according to the manufacturer's protocol. An appropriate amount of RNase free water was then added, depending on the sample amount. To perform cDNA synthesis and real-time PCR (RT-PCR), the Transcriptor Universal cDNA Master (Roche) and PowerUp SYBR Green Master Mix (ThermoFisher) were used, respectively. As shown in Table 1, all the PCR primer sequences were designed by Invitrogen.

**Table 1:** Primer list.

**Gene**	**Forward primer**	**Reverse primer**	**Amplicon size (bp)**
*Gapdh*	TGTTCCTACCCCCAATGTGT	GGTCCTCAGTGTAGCCCAAG	137
*Runx2*	CAGTCCCAACTTCCTGTGCT	TACCTCTCCGAGGGCTACAA	94
*Osx*	CTCCATCTGCCTGACTCCTT	GGGACTGGAGCCATAGTGAG	91
*Alp*	GCTGATCATTCCCACGTTTT	ACCATATAGGATGGCCGTGA	120
*Col1a1*	CGTGCAATGCAATGAAGAAC	TCCCTCGACTCCTACATCTTCT	118
*Dlx5*	CCAGCCAGAGAAAGAAGTGG	TTGGTTTACCATTCACCATCC	56
*Bsp*	GGAGGCAGAGAACTCCACAC	TTCTGCATCTCCAGCCTTCT	130
*Ocn*	GCGCTCTGTCTCTCTGACCT	CGCCGGAGTCTGTTCACTAC	107
*Opn*	CCCGGTGAAAGTGACTGATT	GGCTTTCATTGGAATTGCTT	191
*Phex*	AGGCATCACATTCACCAACA	ATGGCACCATTGACCCTAAA	103
*Dmp1*	GGTTTTGACCTTGTGGGAAA	AATCACCCGTCCTCTCTTCA	91
*CapG*	TCGGCATTTCACAAGACAAC	GTTGGACTTTCCACCACACC	186
*Sost*	CGTGCCTCATCTGCCTACTT	ATAGGGATGGTGGGGAGGT	185

All the markers shown below have been classified according to the method described in previous studies (Bonewald, 2011; Capulli et al., 2014). We examined runt related transcription factor 2 (*Runx2*) and osterix (*Osx*) for osteoprogenitor markers, while alkaline phosphatase (*Alp*) and collagen type I alpha 1 chain (*Col1a1*) acted as pre-osteoblast markers. To identify osteoblast markers, we examined the gene expression of distal-less homebox 5 (*Dlx5*), bone sialoprotein (*Bsp*), and osteocalcin (*Ocn*). With regard to osteocyte markers, we divided the markers into three groups, depending on osteocyte maturity, as markers of osteoid osteocytes, mineralizing osteocytes, and mature osteocytes. Osteopontin (*Opn*) and the phosphate regulating endopeptidase homolog X-linked (*Phex*) were investigated, to check the gene expression levels of osteoid osteocyte markers. Among mineralizing osteocyte markers, we examined the dentin matrix protein 1 (*Dmp1*), capping actin protein, and gelsolin-like (*CapG*) protein, whereas sclerostin (*Sost*) was considered as a marker of mature osteocytes. All the gene expression levels were normalized to those of glyceraldehyde-3-phosphate dehydrogenase (*Gapdh*), using the 2^−ΔΔ*Ct*^ method.

### Immunostaining

The spheroids were collected and fixed in the 4% paraformaldehyde. After washing samples with PBS, they were subjected to permeabilization using 0.1% triton X-100 for 30 min, after which the samples were washed twice with PBS. Then, the samples were blocked using 4% bovine serum albumin at room temperature for 1 h. After washing the blocking reagent away, we added the anti-DMP1 antibody (Abcam), and incubated the reaction mixture overnight at 4°C. The samples were then washed twice with PBS and treated with the Alexa Flour 546 secondary antibody (Invitrogen), Alexa Fluor 488 Phalloidin (Invitrogen), and DAPI (Sigma) for 1 h at room temperature. After the samples were washed twice with PBS, they were preserved in PBS at 4°C until the time of observation. The stained samples were observed using the FLUOVIEW FV3000 (Olympus).

### Microarray

We prepared RNA samples as described above, via the RT-PCR method. The RNA samples were then examined using a spectrophotometer, to check their purity. After reverse transcription, followed by hybridization, the samples were analyzed by using the GeneChip Array (Mouse Genome 430 2.0 Array), which is comprised of 34,000 genes.

In the individual experimental set, the raw data obtained using the spheroid samples were normalized to the monolayer sample. Relative gene expression levels were more than doubled or halved for some samples; these changes were considered to be modulated. Moreover, we only included samples whose *p*-value was lower than 0.05. By sorting those modulated genes, we categorized and ranked them to assess the significance of modulated gene expression levels. To generate a heatmap of gene expression changes in the monolayer and spheroids, we utilized transcriptome analysis console (TAC) software (Thermo Fisher). From the resources section of DAVID Bioinformatics 6.8, we obtained the expression analysis systematic explorer (EASE) score for the enriched gene ontology according to the relevant biological process, cellular components, or molecular functions.

### Statistical Analysis

The bars in the RT-PCR represent the means ± standard error values. A student's *t*-test, one-way, or two-way ANOVA with Fisher's least significant difference (LSD) *post-hoc* test (with α = 0.05) were performed, to evaluate the statistical significance. If a *p*-value in the student's *t*-test is <0.05, we assumed that the difference was significant.

## Results

### The 3D Bone Spheroid Structure Highly Up-Regulated Osteocyte Markers Without Chemical Supplements

**Figure 1:**
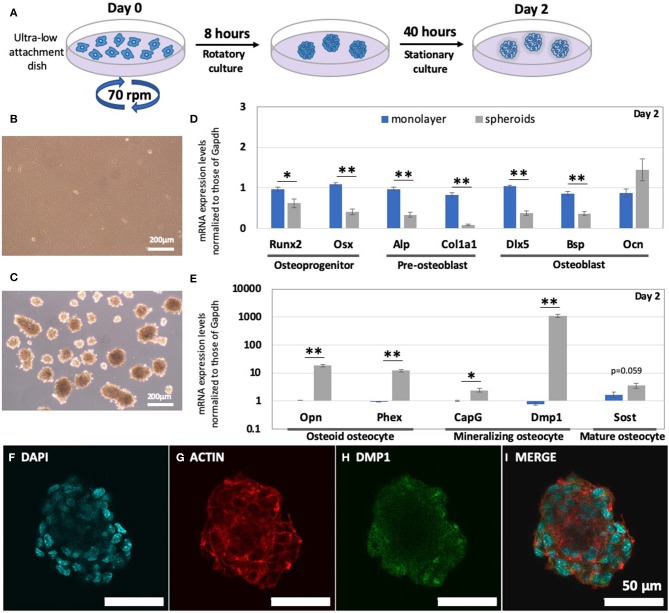
Enhanced levels of osteocyte markers of pre-osteoblast cells, in the form of spheroids. **(A)** Schematic of the method for fabricating bone spheroids using osteoblast precursor cells. Cells were subjected to a rotatory culture process in an ultra-low attachment dish for 8 h, followed by 40 h of the stationary culture process. Morphology of **(B)** monolayer and **(C)** bone spheroids after a 2-days incubation period. **(D)** mRNA expression levels of osteoprogenitor, pre-osteoblast, and osteoblast markers (*Runx2, Osx, Alp, Col1a1, Dlx5, Bsp*, and *Ocn*) in the monolayer and spheroids were measured by RT-PCR. **(E)** Osteocyte mRNA expression levels (*Opn, Phex, CapG, Dmp1*, and *Sost*) in the monolayer and spheroids were measured by RT-PCR. Graphs show that the mRNA expression levels were normalized to the *Gapdh* mRNA expression level. The bars represent the mean ± standard error (*n* = 7; *p*-value was obtained from student's *t*-test; *p < 0.05, **p < 0.005). Images after the staining of bone spheroids, 2 days after cultivation; **(F)** DAPI; **(G)** ACTIN; **(H)** DMP1; **(I)** MERGE.

Based on a previous study (Furukawa et al., 2001), we successfully established the method for fabricating bone spheroids using mouse pre-osteoblastic MC3T3-E1 cells, as illustrated in Figure 1A. After a 2-days incubation period, the monolayer and spheroids reconstructed using MC3TE-E1 cells were formed, as shown in Figures 1B,C. In Figures 1D,E and Table S1, their gene expression levels were then examined via RT-PCR. Osteoprogenitor, pre-osteoblast, and osteoblast markers between the monolayer and spheroids were compared in Figure 1D. In the spheroids, all these markers were significantly down-regulated, as compared to those in the monolayer condition, with the exception of *Ocn*. The osteoprogenitor markers *Runx2* (0.64-fold change; *p* < 0.05) and *Osx* (0.37-fold change; *p* < 0.005) were significantly down-regulated. Moreover, the changes in the pre-osteoblast markers, such as *Alp* (0.35-fold change; *p* < 0.005) and *Col1a1* (0.11-fold change; *p* < 0.005) were also greatly decreased. Among the osteoblast markers, we examined the changes in the expression of *Dlx5, Bsp*, and *Ocn*. Whereas, the relative expression of *Dlx5* (0.37-fold change; *p* < 0.005) and *Bsp* (0.43-fold change; *p* < 0.005) was significantly suppressed, the *Ocn* gene expression (1.65-fold change; *p* = 0.26) was increased, though the level of increase was non-significant.

Conversely, osteocyte markers in the spheroids were greatly up-regulated, as compared to those in the monolayer, as represented in Figure 1E. The relative gene expression levels of osteoid osteocyte markers, including *Opn* (17.5-fold change; *p* < 0.005) and *Phex* (13.0-fold change; *p* < 0.005), were highly up-regulated. The spheroid structure also significantly up-regulated mineralizing osteocyte markers, such as *CapG* (2.37-fold change; *p* < 0.05) and *Dmp1* (1,390-fold change; *p* < 0.005). With regard to mature osteocyte markers, we also evaluated the genetic expression of *Sost*, and the relative change in its levels was up-regulated (2.13-fold change; *p* = 0.059). In addition, we conducted an immunofluorescence analysis of the spheroids in Figures 1F–I, to determine the levels of expression of the osteocyte protein DMP1. As a result, the DMP1 protein expression levels inside the spheroid was confirmed within 2 days.

### Structural Effects on Osteocyte Markers Were Much Greater and Were Observed More Rapidly Than the Effects Attributable to Chemical Induction

**Figure 2:**
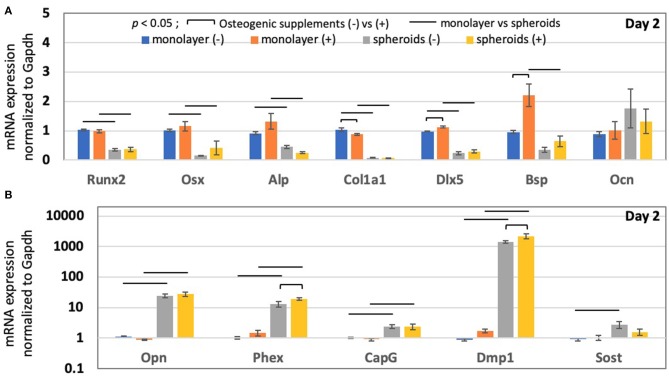
The mRNA expression in the monolayer and spheroids after a 2-days incubation period, in the absence and presence of osteogenic supplements, including ascorbic acid and β-glycerophosphate. **(A)** Osteoprogenitor, pre-osteoblast and osteoblast mRNA expression (*Runx2, Osx, Alp, Col1a1, Dlx5, Bsp*, and *Ocn*), and **(B)** osteocyte mRNA expression (*Opn, Phex, CapG, Dmp1*, and *Sost*) in the monolayer and spheroids were measured by RT-PCR, in the absence and presence of osteogenic supplements, respectively. Graphs show that the mRNA expression levels were normalized to the *Gapdh* mRNA expression level. The colored bars represent the mean ± standard error (*n* = 4; bar indicates the significance between the groups, which was derived via two-way ANOVA and Fisher's LSD *post-hoc* test; α = 0.05).

To evaluate the effects attributable to cell structure on pre-osteoblastic cells, as compared to those attributable to the use of chemicals, we included osteogenic supplements during cultivation and carried out RT-PCR. Figure 2A shows that the addition of osteogenic supplements in the monolayer for 2 days did not affect *Runx2* (Two-way ANOVA: *p* < 0.005 for monolayer vs. spheroids; *p* = 0.96 for control vs. osteogenic supplements; *p* = 0.40 for interaction), *Osx* (Two-way ANOVA: *p* < 0.005 for monolayer vs. spheroids; *p* = 0.16 for control vs. osteogenic supplements; *p* = 0.68 for interaction), *Alp* (Two-way ANOVA: *p* < 0.005 for monolayer vs. spheroids; *p* = 0.46 for control vs. osteogenic supplements; *p* = 0.05 for interaction), and *Ocn* (Two-way ANOVA: *p* = 0.61 for monolayer vs. spheroids; *p* = 0.82 for control vs. osteogenic supplements; *p* = 0.78 for interaction) expression. However, the addition of chemical osteogenic supplements in the monolayer for 2 days slightly decreased *Col1a1* mRNA expression (Two-way ANOVA: *p* < 0.005 for monolayer vs. spheroids; *p* < 0.05 for control vs. osteogenic supplements; *p* = 0.06 for interaction) and significantly up-regulated the expression of osteoblast markers, such as *Dlx5* (Two-way ANOVA: *p* < 0.005 for monolayer vs. spheroids; *p* < 0.05 for control vs. osteogenic supplements; *p* = 0.28 for interaction) and *Bsp* (Two-way ANOVA: *p* < 0.005 for monolayer vs. spheroids; *p* < 0.05 for control vs. osteogenic supplements; *p* < 0.05 for interaction). However, the addition of osteogenic supplements to the spheroids did not cause a significant change in the genetic expression of osteoprogenitor, pre-osteoblast, and osteoblast markers.

In Figure 2B, the osteocyte markers were then analyzed. The addition of osteogenic supplements did not significantly alter the genetic expression of osteocyte marker genes in the monolayer, such as *Opn* (Two-way ANOVA: *p* < 0.005 for monolayer vs. spheroids; *p* = 0.64 for control vs. osteogenic supplements; *p* = 0.58 for interaction), *Phex* (Two-way ANOVA: *p* < 0.005 for monolayer vs. spheroids; *p* = 0.09 for control vs. osteogenic supplements; *p* = 0.13 for interaction), *CapG* (Two-way ANOVA: *p* < 0.005 for monolayer vs. spheroids; *p* = 0.77 for control vs. osteogenic supplements; *p* = 0.94 for interaction), *Dmp1* (Two way ANOVA: *p* < 0.005 for monolayer vs. spheroids; *p* = 0.11 for control vs. osteogenic supplements; *p* = 0.11 for interaction), and *Sost* (Two-way ANOVA: *p* < 0.05 for monolayer vs. spheroids; *p* = 0.19 for control vs. osteogenic supplements; *p* = 0.12 for interaction). However, on day 2, the spheroid samples were subjected to chemical induction, which resulted in the up-regulation of several osteocyte markers, including *Phex* (1.45-fold change; *p* < 0.05) and *Dmp1* (1.53-fold change; *p* < 0.05). Nonetheless, the results altogether showed that the changes in osteocyte gene expression induced by chemical induction were much less than those induced because of the structural effects.

### Cell Condensation in the 3D Structure Is Essential for Continued Osteocyte Differentiation

**Figure 3:**
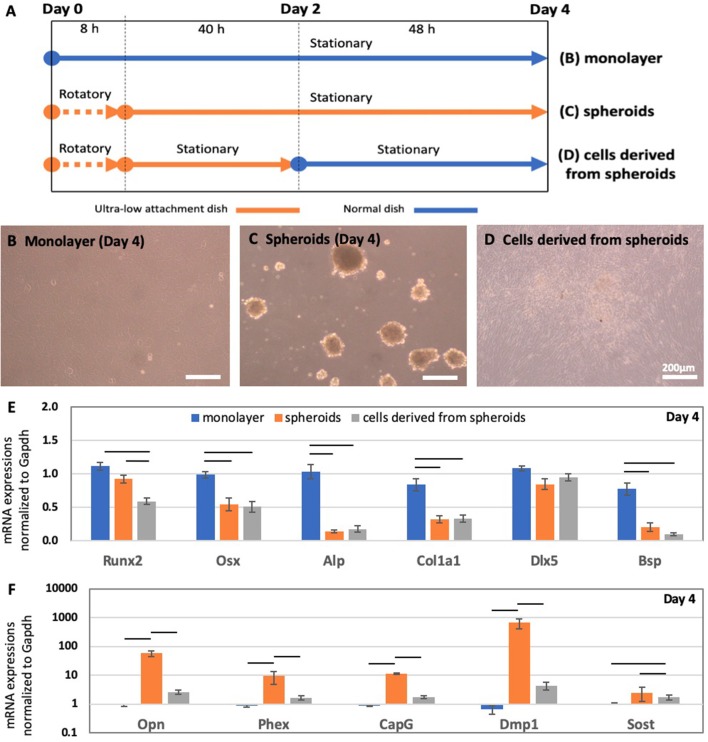
**(A)** Schematic of the experimental timeline for deriving the monolayer, spheroids, and spheroid-derived cells after a 4-days incubation period. Morphology of osteoblast precursor cells in the form of the **(B)** monolayer and **(C)** spheroids after a 4-days incubation period. **(D)** Cells derived from spheroids, after spheroids were transferred onto a culture dish on day 2. After incubating them for 2 days in the ultra-low attachment dish, the spheroids were transferred into a culture dish and incubated further for 2 days. RT-PCR analysis of the mRNA expression of **(E)** pre-osteoblast and osteoblast markers (*Runx2, Osx, Alp, Col1a1, Dlx5*, and *Bsp*) and **(F)** osteocyte markers (*Opn, Phex, CapG, Dmp1, and Sost*) in the monolayer, spheroids, and spheroid-derived cells. Graphs show that the mRNA expression levels were normalized to *Gapdh* mRNA expression levels. The colored bars represent the mean ± standard error (*n* = 6; bar indicates the significance between the groups, which was derived via one-way ANOVA with Fisher's LSD *post-hoc* test; α = 0.05).

To evaluate the significance of the cell condensation induced by the 3D spheroid structure on the MC3T3-E1 cells, we introduced a new model, i.e., cells derived from the spheroids. To prepare this model, spheroids were cultured in the ultra-low attachment dish for 2 days, and then transferred to a standard culture dish, as described in Figure 3A. In comparison with this model, we utilized the 2D monolayer and 3D spheroids incubated for 4 days, which are shown in Figures 3B,C, respectively. The cells derived from spheroids became attached onto the standard culture dish and became spread out over the culture dish immediately after the spheroids were transferred to the culture dish. After a 2-days period of stationary culture, the spheroids almost lost their 3D structure and formed a 2D monolayer, as described in Figure 3D.

As shown by RT-PCR in Figures 3E,F, the gene expression levels of osteoprogenitor [*Osx* (One way ANOVA: *p* < 0.005)], pre-osteoblast [*Alp* (One way ANOVA: *p* < 0.005) & *Col1a1* (One way ANOVA: *p* < 0.005)], and osteoblast markers [*Dlx5* (One way ANOVA: *p* = 0.16) & *Bsp* (One way ANOVA: *p* < 0.005)] in cells derived from the spheroids were similar level to those for spheroids incubated for 4 days. However, osteocyte markers, including *Opn* (One way ANOVA: *p* < 0.005), *Phex* (One way ANOVA: *p* < 0.05), *CapG* (One way ANOVA: *p* < 0.005), *Dmp1* (One way ANOVA: *p* < 0.05), and *Sost* (One way ANOVA: *p* < 0.05) showed were down-regulated up to the level of the monolayer in cells derived from the spheroids.

### Cell Condensation Achieved From the 3D Spheroid Structure Modulated the Clustering of Enriched Gene Expression During the ECM-Receptor Interaction, Cell Proliferation, Cell Cycle, Hypoxia, and Ossification

**Figure 4:**
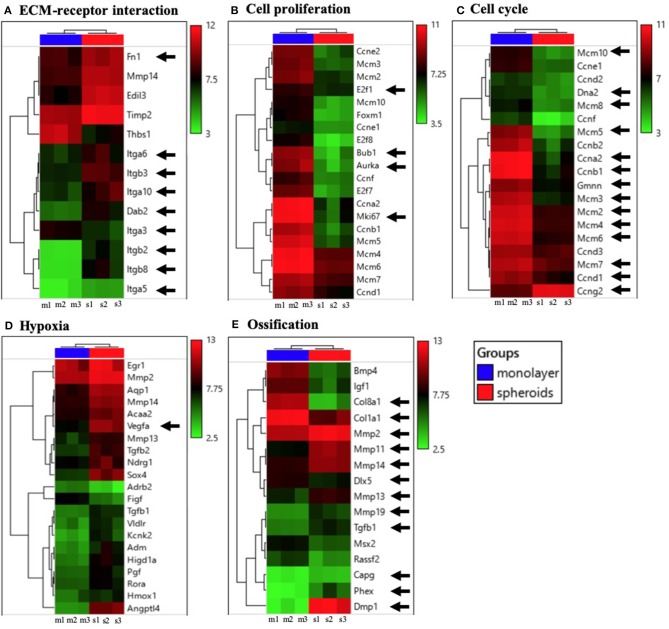
Heatmaps of significantly modulated genes enriched in **(A)** ECM-receptor interaction, **(B)** cell proliferation, **(C)** cell cycle, **(D)** hypoxia, and **(E)** ossification. The relative gene expression levels, for which the fold-changes were above 2.0 or below 0.5, and whose *p*-value was lower than 0.05, were selected (*n* = 3).

In order to investigate all the changes in gene expression in the spheroids, as compared to those in the monolayer, we performed a microarray analysis. Figure 4 shows the heatmap of mRNAs, in accordance with the gene ontology, (A) ECM-receptor interaction, (B) cell proliferation, (C) cell cycle, (D) hypoxia, and (E) ossification. ECM-receptor interaction was significantly activated in the spheroids, as shown in Figure 4A. The results showed that the enrichment of ECM occurred because the 3D structure caused significant changes in integrins (*Itga6, Itgb3, Itga10, Itga3, Itgb2, Itgb8, Itga5*) as well as the up-regulation of *Fn1* or *Dab2*.

The expression of many cell proliferation-related genes, including *E2f1, Bub1, Aurka*, and *Mki67* was significantly suppressed in spheroids, as shown in Figure 4B. With regard to the gene ontology (GO) term, over-represented categories of functional genes were ranked by their EASE scores (gene-enrichment), as shown in Table S2. The highest and second highest EASE scores represented cell cycle and cell division, respectively. A significant down-regulation of DNA replication-related genes (*Dna2, Ccna2, Gmnn*, etc.), M phase-related genes (*Ccnb1*), and G1 phase-related genes (*Mcm10, Mcm8, Mcm5, Mcm3, Mcm2, Mcm4, Mcm6*, etc.) was observed, as shown in Figure 4C, whereas cell expression levels of cycle arrest (G1/G2 phase arrest) genes, including *Ccng2*, were significantly altered. In addition, hypoxia-related genes, including *Vegf* in Figure 4D were significantly modulated in the spheroids, as compared to the level of modulation observed in the monolayer. The ossification markers including the osteocyte markers, matrix metalloproteinase (*Mmp*) family (Prideaux et al., 2015), and *Tgfb1* (Janssens et al., 2005) are shown in Figure 4E. They resulted in the up-regulation of osteocyte-related markers (*Mmp2, Mmp11, Mmp14, Mmp13, Mmp19, Tgfb1, CapG, Phex, Dmp1*, etc.) as well as down-regulation of osteoblast-related markers (*Col8a1, Col1a1, Dlx5, Igf1*, etc.), which was consistent with the RT-PCR results. The genes that were the most significantly up- and down-regulated in the spheroids, as compared to the monolayer were *Dmp1* and *Col8a1*, respectively.

## Discussion

In this study, we successfully developed the 3D cell structures using mouse pre-osteoblast cells, known as bone spheroids. Because we hypothesized that the cell condensation plays a significant role in osteogenesis differentiation, we utilized 3D spheroid models, which enabled us to evoke the cell condensation. As a result, we found out that osteogenic differentiation into osteocyte-like cells in the spheroid was remarkably promoted, as compared to that in the 2D monolayer model, without using chemical supplements. In Figure 1, most of the pre-osteoblast or osteoblast markers in spheroids were significantly suppressed in 2 days, whereas the osteocyte markers (*Opn, Phex, CapG, Dmp1*, and *Sost*) were highly up-regulated. The increase in *Dmp1* gene expression was especially significant, as it was an ~1,400-fold change, as shown by both RT-PCR and microarray analysis, because *Dmp1* is regarded as an osteocyte specific marker that modulates osteocyte formation (Kalajzic et al., 2004; Lu et al., 2011). This level of change in *Dmp1* gene expression, achieved in the form of spheroids, has not been reported with the conventional 2D model, even after long periods of cell culture with chemical supplements. Moreover, the results of immunostaining analysis confirmed the level of expression of the DMP1 protein in the spheroid. Since the matured osteocyte marker, *Sost*, was modestly up-regulated, pre-osteoblast cells in the spheroids appeared similar to cells at a more immature stage of osteocyte differentiation. Nevertheless, it is still satisfactory to note that our model enables us to trigger and accelerate the *in vitro* process of osteocyte differentiation within a short period of 2 days, which is greater than that for the chemical induction process.

With regard to the process of bone nodule formation observed in the 2D *in vitro* model (Alami et al., 2016; Kawai et al., 2019), it is known that its 3D dome-shaped structure was formed after the subcultured cells became confluent and led to the cell condensation. As shown in a previous study (Maniatopoulos et al., 1988), the osteocyte markers derived from pre-osteoblasts to which osteogenic supplements were added were expressed only inside the bone nodule over 3–4 weeks. The up-regulation of osteomarkers in the spheroids seemed to be a similar phenomenon induced by highly condensed cell culture conditions. This suggested that each of the spheroids in our model might mimic the bone nodule. Interestingly, our model did not even require any chemical induction for the significant changes in the osteoblast and osteocyte markers to occur in the short-term period, within 2 days.

We then carried out the experiments in the presence of the osteogenic supplements, to evaluate the effect of chemical induction on the spheroids and compare the chemical effects to the structural 3D effects. Though osteogenic supplements were added, as shown in Figure 2, the culture period for the monolayer was not enough to induce significant changes in the osteoblast or osteocyte markers within 2 days, although the levels of several osteoblast markers, including *Dlx5* and *Bsp* in the monolayer were slightly increased. However, the osteogenic supplements helped to further promote *Phex* and *Dmp1* gene expression in the spheroids, but the effects attributable to chemicals were not as much as the structural effects caused because of 3D structure formation. These results imply that the osteocyte differentiation of pre-osteoblast cells strongly depends on the 3D structural effect, because the cell condensation was enhanced beyond those observed with chemical induction.

To determine the significance of cell condensation for osteocyte differentiation, we transferred spheroids from the ultra-low attachment dish to a standard culture dish after a 2-days incubation period, and allowed the spheroids to be cultured for an additional 2 days. As a result, the spheroids lost their 3D structure and were converted into a 2D monolayer, as shown in Figure 3. This implies that the spheroids formed for 2 days in the ultra-low attachment dish were still active enough for the cell-substrate interaction to occur, for their attachment to the standard plastic cell culture dish. Interestingly, cells derived from the spheroids were spread out over the culture dish, and the expression of their osteocyte genes as spheroids was reversed to the level of a monolayer cultured for 4 days. Hence, the up-regulation of osteocyte genes of pre-osteoblast cells can be maintained, when the cells exhibit the cell condensation observed in the 3D structure. Thus, osteocyte differentiation from pre-osteoblast or osteoblast cells was triggered by the cell condensation, whereas cells at lower cell condensation levels become more inactive with regard to the induction of osteocyte differentiation.

Due to the structural environment of spheroids, the cell condensation for pre-osteoblast cells was induced inside the spheroids, and this eventually brought about an increase in the ECM secreted by surrounding cells. Because of microarray analysis in Figure 4, ECM accumulation induced intracellular signaling pathways in the surrounded cells via integrins. The results of microarray analysis also showed that the cell condensation achieved in the spheroid caused a cessation in cell proliferation and simultaneously allowed cells to induce cell cycle arrest. These events altogether might cause the pre-osteoblast cells in the spheroids to become terminally differentiated into osteocyte-like cells. Moreover, hypoxia was thought to contribute to the promotion of osteocyte differentiation inside spheroids, as hypoxic conditions are known to induce osteocytogenesis differentiation (Hirao et al., 2007; Zahm et al., 2008). The up-regulation of hypoxia-related markers, including *Vegf* was confirmed in the spheroids. This occurred because the cells inside the spheroid were subjected to hypoxic conditions instead of monolayer conditions, which enhanced the up-regulation of osteocyte markers further. We think that several potential factors could induce the osteocyte differentiation after cell condensation; however, further studies, including those regarding the cell-cell interaction force, cytoskeletal tension, or cell shape would be required to understand the mechanism in detail.

**Figure 5:**
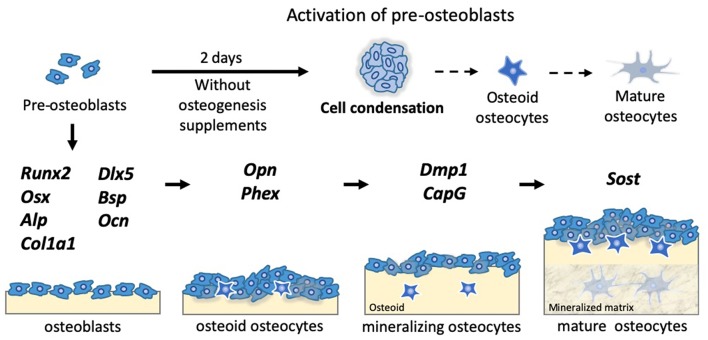
Schematic diagram illustrating the significance of the highly condensed condition of osteoblast precursor cells differentiating into osteocytes *in vitro* and *in vivo*.

As illustrated in Figure 5, we showed that it is more efficient and essential for pre-osteoblast cells in the highly condensed 3D structure to trigger and facilitate differentiation into osteocyte-like cells *in vitro*. To date, the osteoblast is only thought to line up two-dimensionally on the bone at a targeted site, where the bone remodeling occurs *in vivo*. Our results altogether suggest that osteoblasts achieve the ability to differentiate into osteocyte-like cells, after they proliferate and become condensed at the targeted site of bone remodeling. For several decades, researchers have attempted to induce osteocyte differentiation over 1–2 months, by culturing cells to ensure that they are over-confluent and condensed, until the formation of the 3D bone nodule, in combination with chemical supplements (Bhargava et al., 1988; Maniatopoulos et al., 1988; Schecroun and Delloye, 2003). However, this study showed that osteocyte differentiation highly depends upon cell condensation levels that are beyond those attributable to a longer period of chemical induction. Although it is extremely difficult to observe this event *in vivo*, its occurrence would help us to narrow the gap between *in vitro* and *in vivo* bone remodeling. Hence, we first highlighted the significance of a highly condensed 3D structure, in which pre-osteoblast or osteoblast cells would be induced to differentiate into osteocyte-like cells. Our group has also developed a 3D tissue engineered construct of a different type, which was reconstructed using only pre-osteoblast cells (MC3T3-E1), without the introduction of any biomaterial. It rendered the changes in gene expression levels similar to those observed in the present study, with regard to the up-regulation in the osteocyte marker levels and down-regulation in the osteoblast marker levels. These results together reveal the significance of cell condensation, and the manner in which the pre-osteoblast cells are triggered to undergo osteocyte differentiation. In this study, we utilized murine osteoblast precursor cells, which are well-studied and widely used cell lines in bone research; however, further research needs to be conducted to support our findings using a different cell line or primary cells, such as stem cells. Moreover, it will be meaningful to carry out experiments involving bone spheroids over a longer period, to determine further changes in their functioning or histology as osteocytes.

Here, we first established a novel method to fabricate 3D bone spheroids using mouse precursor osteoblast cells under rotatory culture conditions, by changing several parameters, including the cell density or rotational speed, and the time set for the rotatory shaker, based on the parameters described in the previous study (Furukawa et al., 2001). Although several studies attempted to fabricate the 3D *in vitro* structure for generating the bone model that enhanced osteogenic differentiation, they utilized scaffold-based techniques, and used biomaterials, such as collagen or agar-agar gel (Restle et al., 2015; Zujur et al., 2017). Moreover, these models could slightly enhance osteocyte differentiation with the accompanying process of osteoblast differentiation, because of which the factor triggering independent osteocyte differentiation remained unknown. For biomedical applications using a tissue engineering approach, these mediating materials might carry the risk of affecting cell behavior or inducing negative interactions with donor cells after transplantation. Because our bone spheroids did not recruit any artificial materials during the fabrication process, they are predicted to become a more appropriate model for clinical use. Particularly, our method allowed the mass production of spheroids, whereas spheroids are conventionally generated one by one in a tube or well. This method can be optimized and applied to fabricate spheroids for various cell types, by adjusting the cell density or rotatory culture period, because the strength of cell-cell interactions or cell-ECM interactions differ for varied types of cells. Furthermore, the 3D *in vitro* structures for other cell models could enhance their differentiation capability, as shown in this study.

In the field of bone tissue engineering, mesenchymal stem cells (MSC), embryonic stem cells (ESC), or induced pluripotent stem cell (iPSC) reportedly participate in the repair of osseous defects, such as traumatic fractures, osteoarthritis, or rheumatoid arthritis (Amini et al., 2012; Goonoo and Bhaw-Luximon, 2018). Moreover, MSC treatment for large bone defects or the severe osteogenesis imperfecta model was provided in several studies (Horwitz et al., 1999; Quarto et al., 2009). However, the limited differentiation capacity of MSCs remains a concern observed for current clinical therapies, whereas the utilization of more differentiated cells is thought to be safer. The use of our model allows the mass production of differentiated spheroids *in vitro*, in combination with osteogenic supplements, and further enables us to transplant them to a targeted site via a syringe injection. Several applications of spheroids can be expected to be developed in the bone tissue engineering field.

In conclusion, this study provides new insights into the significance of the cell condensation conditions achieved via the 3D structure, to enable the osteoblast precursor cells to facilitate differentiation into osteocyte-like cells. Our 3D bone spheroid model, which was generated using mouse osteoblast precursor cells, demonstrated the significant up-regulations in the osteocyte markers within 2 days. Consequently, we suggested that cell condensation is the key factor triggering greater levels of *in vitro* osteocyte differentiation than that observed with chemical induction.

## Data Availability Statement

All datasets generated for this study are included in the manuscript/Supplementary Files.

## Author Contributions

JK and TA contributed to the study design, data collection, manuscript writing, and final approval of the manuscript.

### Conflict of Interest

The authors declare that the research was conducted in the absence of any commercial or financial relationships that could be construed as a potential conflict of interest.

## Supplementary Material

Table S1 Expression levels of genes in monolayer and spheroids after 2 days cultivation. The average Ct values were exhibited in the table.

Table S2 Over-represented gene functional categories with EASE scores (gene-enrichment).
