# Supplementary figures and images for: Cell Condensation Triggers the Differentiation of Osteoblast Precursor Cells to Osteocyte-Like Cells
Source: Front Bioeng Biotechnol. 2019 Oct 23;7:288. doi: 10.3389/fbioe.2019.00288 (PMC6819367; doi:10.3389/fbioe.2019.00288)

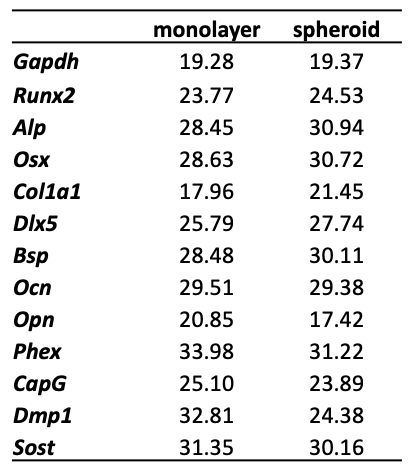

Supplement: Table S1 — Expression levels of genes in monolayer and spheroids after 2 days cultivation. The average Ct values were exhibited in the table. [file Image_1.JPEG]

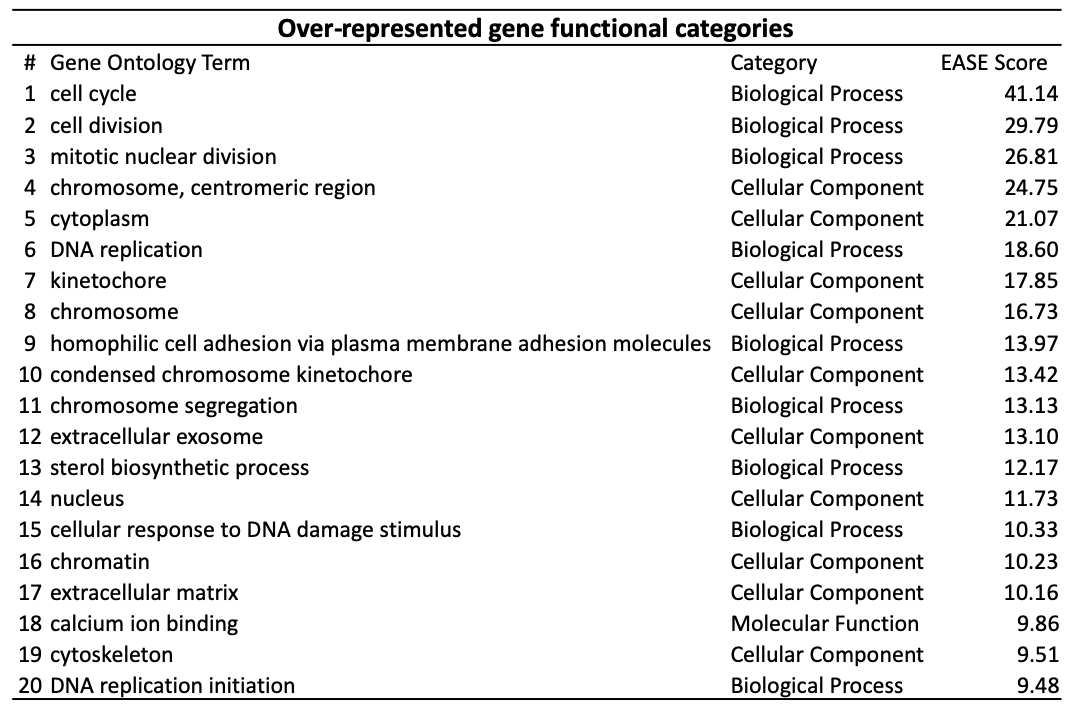

Supplement: Table S2 — Over-represented gene functional categories with EASE scores (gene-enrichment). [file Image_2.JPEG]
